# Efficient and reproducible high resolution spiral myocardial phase velocity mapping of the entire cardiac cycle

**DOI:** 10.1186/1532-429X-15-34

**Published:** 2013-04-15

**Authors:** Robin Simpson, Jennifer Keegan, David Firmin

**Affiliations:** 1 NIHR Royal Brompton Cardiovascular Biomedical Research Unit, London, UK; 2 Imperial College, London, UK; 3 Cardiovascular Magnetic Resonance Unit, Royal Brompton Hospital, Sydney Street, London, SW3 6NP, UK

**Keywords:** Cardiovascular magnetic resonance, Phase velocity mapping, Spiral, Retrospective cardiac gating, Regional myocardial motion

## Abstract

**Background:**

Three-directional phase velocity mapping (PVM) is capable of measuring longitudinal, radial and circumferential regional myocardial velocities. Current techniques use Cartesian k-space coverage and navigator-gated high spatial and high temporal resolution acquisitions are long. In addition, prospective ECG-gating means that analysis of the full cardiac cycle is not possible. The aim of this study is to develop a high temporal and high spatial resolution PVM technique using efficient spiral k-space coverage and retrospective ECG-gating. Detailed analysis of regional motion over the entire cardiac cycle, including atrial systole for the first time using MR, is presented in 10 healthy volunteers together with a comprehensive assessment of reproducibility.

**Methods:**

A navigator-gated high temporal (21 ms) and spatial (1.4 × 1.4 mm) resolution spiral PVM sequence was developed, acquiring three-directional velocities in 53 heartbeats (100% respiratory-gating efficiency). Basal, mid and apical short-axis slices were acquired in 10 healthy volunteers on two occasions. Regional and transmural early systolic, early diastolic and atrial systolic peak longitudinal, radial and circumferential velocities were measured, together with the times to those peaks (TTPs). Reproducibilities were determined as mean ± SD of the signed differences between measurements made from acquisitions performed on the two days.

**Results:**

All slices were acquired in all volunteers on both occasions with good image quality. The high temporal resolution allowed consistent detection of fine features of motion, while the high spatial resolution allowed the detection of statistically significant regional and transmural differences in motion. Colour plots showing the regional variations in velocity over the entire cardiac cycle enable rapid interpretation of the regional motion within any given slice. The reproducibility of peak velocities was high with the reproducibility of early systolic, early diastolic and atrial systolic peak radial velocities in the mid slice (for example) being −0.01 ± 0.36, 0.20 ± 0.56 and 0.14 ± 0.42 cm/s respectively. Reproducibility of the corresponding TTP values, when normalised to a fixed systolic and diastolic length, was also high (−13.8 ± 27.4, 1.3 ± 21.3 and 3.0 ± 10.9 ms for early systolic, early diastolic and atrial systolic respectively).

**Conclusions:**

Retrospectively gated spiral PVM is an efficient and reproducible method of acquiring 3-directional, high resolution velocity data throughout the entire cardiac cycle, including atrial systole.

## Background

Cardiovascular Magnetic Resonance (CMR) is a well established tool for measuring cardiac function. Global parameters of function such as ejection fraction and ventricular volumes are routinely used clinically as predictors of prognosis [1], however regional dysfunction can be masked by apparently healthy global function in the early stages of disease. Tissue tagging [2,3], displacement encoding with stimulated echoes (DENSE) [4], strain encoding (SENC) [5] and phase velocity mapping (PVM) [6,7] are all capable of measuring regional myocardial motion [8]. While each of these shows great promise for future clinical application, DENSE and SENC have not as yet been extensively validated in large scale trials. In contrast tissue tagging is widely used, however due to tag fading (an issue which also affects DENSE and SENC) it is unable to analyse the entire cardiac cycle (although tag fading can be counteracted by complementary SPAMM techniques).

PVM does not suffer from tag fading and several studies have used PVM to assess regional myocardial motion. Early work measured only longitudinal velocities, but established that the technique was capable of characterising global and regional myocardial motion and detecting abnormal motion in patients with infarcts. However, the long scan times – even though respiratory gating was not used - meant application of the technique was unfeasible in clinical practice [9]. The development of segmented sequences enabled PVM data to be acquired within a breath-hold. Initially, separate breath-holds were required for each direction of encoding (and for the reference scan) [10,11], but the introduction of view-sharing allowed 3-directional velocity mapping (acquired spatial resolution: 3.1 × 1.6 mm, temporal resolution: 60–68 ms) to be completed within a single 16–19 cardiac cycle breath-hold [12]. Breath-hold sequences have since been used to measure regional mechanics in healthy subjects [13,14]. Regional variations in the systolic and diastolic peak velocities and their timings have been shown, together with transmural velocity gradients. However, the relatively low spatial (2.7 × 1.3 mm) and temporal (37–87 ms) resolution achievable with breath-hold techniques is such that fine temporal features in the resulting velocity-time curves cannot be detected and the accuracy of the peak velocity measurements, and the times to those peak velocities, is also compromised.

Improved temporal and spatial resolution may be achieved by acquiring data during free-breathing with diaphragmatic navigators limiting the motion to a small range (typically 5 mm) around the end expiratory pause position [15]. Navigator-gated phase velocity mapping sequences using respiratory navigators have detected fine features of motion, for example a small biphasic pattern of radial velocities in early diastole [16] or complex patterns of ventricular twisting [17]. Subtle differences in regional motion caused by age and gender have been reported, as well as large differences both in peak velocities and the regional homogeneity of the timings of those peak velocities in disease [18,19]. However, the acquisition durations for these studies is relatively long (128 cardiac cycles with acquired spatial resolution of 2.6 × 1.3 mm and temporal resolution of 13.8 ms (view sharing) [16] or 180 cardiac cycles with acquired spatial resolution of 2.6 × 1.4 mm and temporal resolution of 26 ms [19]) and these are further extended by the inherently poor efficiency (typically 40%) of navigator gating techniques.

One approach to speeding up MR acquisitions is to use a more efficient k-space trajectory, such as spirals [20]. Using spirals potentially allows large reductions in scan time while maintaining spatial and temporal resolution. Spiral PVM has been applied to measuring blood flow velocity (for example in the heart [21,22], the coronary arteries [23], renal arteries [24] and deep veins in the calf [25]) but it has not previously been applied to measuring myocardial velocities.

Prospective cardiac gating has been used in all previous PVM studies of the myocardium which limits data acquisition to approximately 90% of the cardiac cycle. In contrast, applying retrospective cardiac gating to PVM of the myocardium would allow the analysis of 100% of the cardiac cycle, including atrial systole, a feature of cardiac motion that to our knowledge has not previously been shown with MR studies. In healthy motion atrial systole accounts for 20–30% of LV filling, but in diseases where diastolic function is impaired, atrial systole can become more important to allow preservation of LV function [26]. Analysis of atrial systole by PVM could therefore be clinically important.

The aim of this work is to develop a spiral trajectory phase velocity mapping sequence allowing the acquisition of high temporal and spatial resolution data in a reasonable time period. The sequence is implemented with retrospective cardiac gating to allow analysis of the entire cardiac cycle. Data are acquired in 10 healthy subjects and global, regional and transmural motion characterised in terms of systolic and early diastolic peak velocities and their timings. In addition, repeat scanning of the ten subjects on a second occasion enables a comprehensive assessment of the inter-study reproducibilities of the global and regional parameters characterising myocardial motion.

## Methods

A retro-gated phase velocity mapping sequence with three orthogonal directions of velocity encoding was developed using spiral readout gradients. K-space was covered using 13 spiral interleaves of 12 ms duration. To improve the temporal resolution of the sequence, reference and velocity encoded data were acquired on consecutive cardiac cycles following a single dummy cardiac cycle. The total acquisition duration for the four datasets making up a single acquisition (reference and 3-velocity encoded) was therefore 53 cardiac cycles (assuming 100% respiratory efficiency). Bipolar velocity encoding gradients prior to the spiral readouts resulted in velocity sensitivities of 30 cm/s through-plane and 20 cm/s in-plane. A black blood pulse of duration 6 ms [13] was output on every third sequence repeat to ensure good blood-myocardium contrast and to reduce any artefacts due to beat-to-beat variations in blood flow [27].

A 1–1 binomial water excitation pulse (flip angle = 15 degrees) was implemented to reduce off-resonance blurring of fat. The acquired temporal resolution was 21 ms. The retrospective reconstruction mode available on the scanner was used to interpolate linearly between the acquired time points to produce 60 equally spaced phases through the heart cycle, leading to a reconstructed temporal resolution of 14–20 ms depending on heart-rate. A field of view of 360 mm was used and the spiral data re-gridded onto a 256 × 256 matrix using a standard gridding technique [28] leading to an acquired spatial resolution of 1.4 × 1.4 mm which was then interpolated to 0.7 mm × 0.7 mm through zero-filling k-space. The slice thickness was 8 mm.

A 90°/180° crossed-pair navigator [29] (duration 9 ms + 10 ms feedback time) positioned over the dome of the right hemi-diaphragm was output at the start of each cardiac cycle and a 5 mm acceptance window positioned over the end expiratory pause position. Preliminary *in vivo* studies showed that a simple accept/reject algorithm based on this single navigator did not provide good respiratory motion compensation throughout the entire cardiac cycle (as discussed later). Instead, the algorithm implemented ensured that data acquired within a given cardiac cycle was only accepted if both the navigators immediately before and after that data acquisition were within the acceptance window. This stricter acceptance criterion - requiring the navigators on two consecutive cardiac cycles to be within the acceptance window - results in markedly reduced scan efficiency and so a guided breathing technique was introduced to ensure reasonable scan duration. During the data acquisition, the respiratory trace was displayed in real-time on a screen visible to the subject in the scanner. The subject could then use this visual feedback to guide their breathing, completing the scan in a series of breath-holds or by slow and steady breathing, or by a combination of the two.

### In vivo studies

The retrogated spiral phase velocity mapping sequence was implemented on a clinical scanner (3 T, MAGNETOM Skyra, Siemens AG Healthcare Sector, Germany) equipped with an anterior cardiac 18-element matrix coil and a 48 element spine array. 6 elements of the cardiac coil and 6 of the spine coil were used for all *in vivo* acquisitions. Localised second-order shimming and frequency adjustment based on the signal from a user-defined adjustment box situated over the whole heart was performed to reduce off-resonance effects. Phase velocity maps were acquired in the basal, mid and apical short axis planes (defined as 25%, 50% and 75% of the long axis length of the LV) in 10 healthy volunteers (mean age 31 years, range 24–56). In all 10 subjects, all three acquisitions were repeated on a second occasion (on average 9.7 days later) in order to assess inter-study reproducibility. The two studies were carried out at similar times of day. Heart rates were noted for each acquisition. The study was in compliance with the Helsinki Declaration and was approved by an institutional review committee (East London REC 3). All subjects gave written informed consent.

Background phase errors were determined for each acquired dataset by scanning a large homogeneous stationary phantom (33 cm × 22 cm × 42 cm, gelatine with 5 mM concentration of Gadolinium) using the same sequence parameters and the same slice positions as in the volunteer study. A simulated ECG with the same heart-rate as the subject was used to trigger the acquisitions. The velocity maps from these phantom acquisitions were smoothed using a median filtering technique to reduce noise and then subtracted from the volunteer data on a pixel-by-pixel basis [30].

### Post-processing

Custom software programmed using MATLAB (The Mathworks Inc., Natick, MA, USA) was used to analyse the images. The myocardium was manually segmented in each frame with a spline drawing algorithm. Both the magnitude images and the velocity maps were used to locate the position of the endo- and epicardial borders by eye. Background corrected velocities were transformed into a cylindrical system of co-ordinates natural to the LV anatomy (with a polar axis defined by the anterior LV-RV meeting point and the centre of mass of the slice at each time-point) and the slices were segmented according to the AHA 17 segment model [31], resulting in six segments for the basal and mid slices, and four segments for the apical slices. The myocardium was also segmented radially into epicardial, endocardial and mid layers by dividing the wall thickness into thirds at 360 angular points around the centre of the polar coordinate system. Average longitudinal (motion towards apex defined as positive), radial (motion towards the center of the slice defined as positive) and circumferential (clockwise as viewed from the apex defined as positive) velocities for each of the segments were calculated, as were global velocities for each slice.

**Figure 1:**
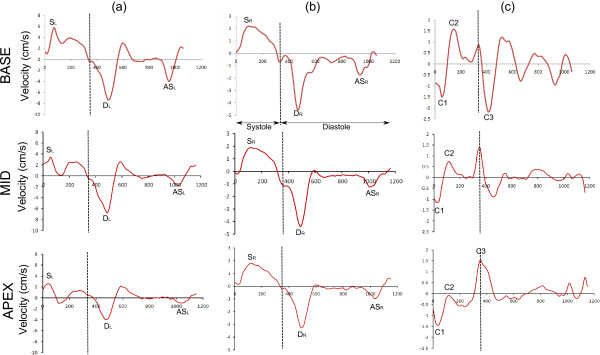
**Global longitudinal (a), radial (b) and circumferential (c) velocity-time curves from the basal, mid and apical slices of a single healthy volunteer.** The longitudinal curves correspond to velocities measured from the longitudinal phase images, whereas the X and Y phase images are combined to produce the radial and circumferential velocities. The length of systole is marked on all curves. The various peaks mentioned in the text are marked. TTP = time to peak, S_L_ = longitudinal peak systole, D_L_ = longitudinal peak diastole, AS_L_ = longitudinal peak atrial systole, S_R_ = radial peak systole, D_R_ = diastolic peak systole, AS_R_ = radial peak atrial systole, C_1_ = first circumferential peak, C_2_ = second circumferential peak, C_3_ = circumferential peak diastole.

Calculated global and segmental parameters are labelled on example basal short-axis longitudinal (a), radial (b) and circumferential (c) velocity-time curves in Figure 1. For both regional and global velocity-time curves, the peak longitudinal and radial systolic (S_L_, S_R_) and diastolic (D_L_, D_R_) velocities were determined together with the times to those peaks (TTP (ms)) from the R-wave. The longitudinal and radial velocity-time curves also show a late peak in atrial systole (AS_L_, AS_R_) which was recorded along with the TTP AS_L_ and TTP AS_R_. In the circumferential velocity-time curves, two early systolic peaks (C1 and C2) were recorded in all three slices along with their TTP values. In the basal and apical slices a third peak (C3) was also seen in early diastole. All TTP values were recorded in ms (from the R-wave) and also as percentages of the length of systole or diastole as appropriate. The length of systole was determined as the time from the R-wave to the first negative radial peak which represented end systole and which could be seen in all scans [16].

In addition, the ratio of the early diastolic peak to the atrial systolic peak in longitudinal and radial directions was calculated for both regional and global velocity-time curves. This is analogous to the parameter E/A (the ratio of peak early (E) filling and late diastolic filling (A) measured at the level of the mitral valve in echocardiography to assess diastolic function [32].

Statistical testing was performed using SPSS (SPSS 19, SPSS, Chicago, Ill). All parameters were checked for normality using the Shapiro-Wilk test. The mean ± standard deviation (SD) of global longitudinal, radial and circumferential parameters was determined for each slice (basal, mid and apical). Repeated measures analysis of variance (with Greenhouse-Geisser correction in the case of non-sphericity) and paired t-testing (with Bonferroni correction for multiple testing) were used to compare parameters between slices. Differences between endo, mid and epi-cardium velocities were similarly analysed.

As well as segmenting the myocardium according to the 17-segment model for the analyses described above, each slice was also segmented into 24 equal angle segments. Regional velocities in these segments were normalised to a fixed RR interval and averaged over the ten healthy volunteers. This normalisation was performed independently for systole, early diastole, diastasis and atrial systole, as discussed later. The velocities were colour coded and displayed in a grid with circumferential position in the myocardium (from anterior to lateral, inferior and septal regions) plotted along the vertical axis and time from the R-wave along the horizontal axis. These 2D colour plots effectively summarise normal regional velocity-time patterns through the entire cardiac cycle.

Inter-study reproducibility of all parameters (peak velocities as well as TTP values) was determined as the mean +/− SD of the signed differences between repeat acquisitions, together with Bland-Altman analysis [33]. The inter-study reproducibility of TTP values was determined both for absolute times (in ms) from the R-wave and for values expressed as a percentage of the length of systole or diastole.

## Results

**Figure 2:**
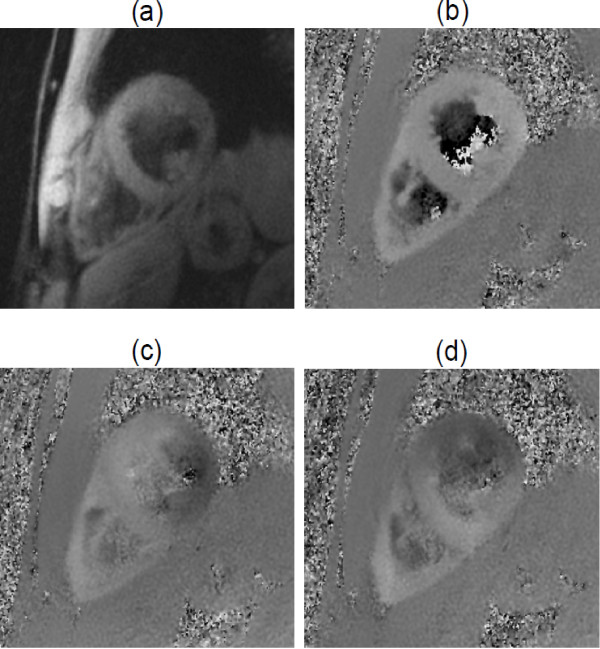
**Example images taken in diastole from the mid-ventricular slice of a single healthy volunteer.** (**a**) shows a magnitude image. The spirals are short enough that there is very little off-resonance blurring and the black blood pulse removes artefact due to beat to beat variations in blood flow. Longitudinal (**b**) and in-plane (**c** and **d**) velocity-encoded images are also shown.

Data were successfully acquired from the basal, mid and apical slices on two separate occasions for all ten healthy volunteers. Figure 2 shows an example diastolic magnitude image of the mid slice of a single volunteer, together with the corresponding through-plane and in-plane velocity maps. The myocardium is clearly depicted, blood and fat suppression is good and the short spiral duration has resulted in there being no major off-resonance blurring. The guided breathing was tolerated well by all subjects with the mean navigator efficiency being 57% (SD ± 9%, range = 38%–82%). The acquisition duration per slice was therefore 95 cardiac cycles (SD ± 16, range =65–138). The average heartbeat duration was 994 ms (SD ± 121 ms, range = 813–1226 ms). The duration of systole was 349 ± 38 ms).

Figure 1 shows example global longitudinal, radial and circumferential velocity-time curves from the basal slice of a single subject. The high temporal resolution allows detailed analysis of small features of the curves, while retrospective cardiac gating allows analysis of the entire cardiac cycle, including atrial systole which has not previously been visualised in magnetic resonance studies. The atrial systolic peak was clearly seen in all global radial curves at all levels, while in longitudinal curves it was seen in all basal and mid curves, and in 17 of the 20 (10 subjects scanned on 2 occasions) apical curves. As expected, the average velocities over the entire cycle for all velocity-time curves were close to zero (values averaged over slices for global velocity-time curves are 0.24 ± 0.28 cm/s for longitudinal velocities, −0.25 ± 0.10 cm/s for radial velocities and 0.043 ± 0.11 cm/s for longitudinal velocities, without phantom subtraction the respective values are 0.55 ± 0.22 cm/s, 0.79 ± 0.42 cm/s and 0.58 ± 0.31 cm/s).

### Normal motion

#### Global velocities

**Figure 3:**
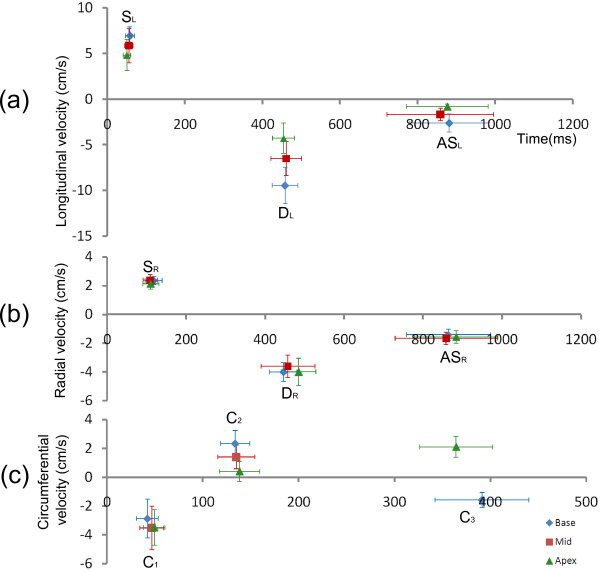
**Mean and standard deviations of peak and TTP velocities for longitudinal (a), radial (b) and circumferential (c) velocities and in all three slices.** Values can be found in Tables 1 and 2.

Mean ± SD of all measured and derived velocity parameters can be found in Table 1 and are shown graphically in Figure 3. All parameters were normally distributed.

**Table 1:** Longitudinal, radial and circumferential peak velocities and E/A values in the basal, mid and apical short-axis slices of 10 healthy volunteers, together with inter-study reproducibility

	**Peak**	**Interstudy reproducibility**
		**Mean ± SD signed differences**
BASE		
S_L_ (cm/s)	6.94 ± 1.87	−0.50 ± 1.07
D_L_(cm/s)	−9.45 ± 1.97	−0.30 ± 1.54
AS_L_(cm/s)	−2.62 ± 0.99	−0.26 ± 1.04
E/A_L_	4.26 ± 2.39	−0.20 ± 1.90
S_R_(cm/s)	2.35 ± 0.28	0.05 ± 0.27
D_R_(cm/s)	−4.01 ±0.65	−0.20 ± 0.96
AS_R_(cm/s)	−1.41 ±0.41	−0.15 ± 0.36
E/A_R_	3.00 ± 0.76	−0.08 ± 0.61
C_1_(cm/s)	−2.88 ± 1.35	0.12 ± 0.58
C_2_(cm/s)	2.33 ± 0.91	0.07 ± 0.64
C_3_(cm/s)	−1.58 ± 0.52	−0.37 ± 0.61
MID		
S_L_ (cm/s)	5.87 ± 1.89	−0.56 ± 0.87
D_L_(cm/s)	−6.50 ± 1.85	0.21 ± 1.54
AS_L_(cm/s)	−1.66 ± 0.67	0.22 ± 1.08
E/A_L_	4.97 ± 3.29	−1.37 ± 4.95
S_R_(cm/s)	2.38 ± 0.38	−0.01 ± 0.36
D_R_(cm/s)	−3.61 ± 0.77	0.20 ± 0.56
AS_R_(cm/s)	−1.59 ± 0.43	0.14 ± 0.42
E/A_R_	2.43 ± 0.87	−0.01 ± 0.66
C_1_(cm/s)	−3.52 ± 1.50	0.10 ± 0.50
C_2_(cm/s)	1.40 ± 0.83	−0.20 ± 0.48
APEX		
S_L_ (cm/s)	4.83 ± 1.70	−0.11 ± 0.67
D_L_(cm/s)	−4.27 ± 1.67	−0.31 ± 1.35
AS_L_(cm/s)	−0.81 ± 0.29 *	0.29 ± 0.78 **
E/A_L_	5.59 ± 3.04*	0.30 ± 0.78 **
S_R_(cm/s)	2.14 ± 0.27	0.02 ± 0.33
D_R_(cm/s)	−3.99 ± 0.94	−0.10 ± 0.95
AS_R_(cm/s)	−1.57 ± 0.45	−0.15 ± 0.48
E/A_R_	2.67 ± 0.66	−0.23 ± 0.64
C_1_(cm/s)	−3.50 ± 1.23	−0.04 ± 0.57
C_2_(cm/s)	0.39 ± 0.69	−0.00 ± 0.38
C_3_(cm/s)	2.09 ± 0.72	0.17 ± 0.59

All values shown are mean ± standard deviation.

*1 excluded, **2 excluded since AS peak not clearly seen.

As expected the peak systolic and diastolic velocities in the longitudinal direction are higher at base than apex (6.94 ± 1.87 cm/s vs 4.83 ± 1.70 cm/s, and −9.45 ± 1.97 cm/s vs −4.27 ± 1.67 cm/s respectively, p < 0.001 for both). In 1 apical velocity-time curve, atrial systole was not clearly shown but in the remaining cases, peak longitudinal atrial systolic velocities also showed a significant decrease from base to apex (−2.62 ± 0.99 cm/s vs −0.81 ± 0.29 cm/s, p < 0.001).

Radial velocities do not show any trend when moving from base to apex, with similar peak systolic, diastolic and atrial systolic velocity values at all levels. Atrial systole is seen clearly in all cases.

Circumferential velocity-time curves show two peaks (C1 and C2) in opposite directions in rapid succession at the beginning of systole at all 3 levels. Both C1 and C2 are higher at base than apex (C1 −2.88 ± 1.35 vs −3.50 ± 1.23, p < 0.01, C2 2.33 ± 0.91 vs 0.39 ± 0.69, p < 0.001). The opposite directions of circumferential motion in early diastole (C3 at base vs C3 at apex) indicate the characteristic untwisting of the ventricle during relaxation. In the mid-slice there is no consistent peak in global velocities at early diastole and so no values for C3 in the mid-slice are shown.

Table 2 contains systolic and diastolic TTP values as absolute times in milliseconds from the R-wave (column 2). TTP values are also shown as percentages of systole or diastole respectively (column 4) and, to facilitate a direct comparison with the timings in column 2, in milliseconds based on the average lengths of systole (350 ms) and diastole (650 ms) (column 6). The standard deviation of TTP in milliseconds is small for the systolic parameters (S_L_, S_R_, C_1_ and C_2_) but increases for early diastolic parameters (D_L_, D_R_ and C_3_) and increases further for late diastolic parameters (AS_L_, AS_R_). This is clearly seen in Figure 3 as increasingly wide horizontal error bars as the time from the R-wave increases. However, when expressed as a percentage of the length of diastole, the variability of the diastolic TTP values is reduced, particularly for atrial systole. For example the average ± SD of TTP AS_L_ is 858.5 ± 137.5 ms from the R-wave but 869.4 ± 30.6 ms when expressed in terms of the average systolic and diastolic length.

**Table 2:** Longitudinal, radial and circumferential time to peak (TTP) values in the basal, mid and apical short-axis slices of 10 healthy volunteers, together with inter-study reproducibility

	**TTP (ms)**	**Interstudy Reproducibility (ms) Mean ± SD signed differences**	**TTP % systole/diastole**	**Interstudy Reproducibility (%) Mean ± SD signed differences**	**TTP (ms based on average length of systole and diastole)**	**Interstudy reproducibility, Mean ± SD signed differences**
BASE						
S_L_	58.3 ± 11.8	0 ± 8.1	17.3 ± 1.9	−0.5 ± 1.8	60.6 ± 6.7	−1.8 ± 6.3
D_L_	458.0 ± 33.3	18.7 ± 41.1	18.0 ± 4.0	0.7 ± 4.3	467 ± 26	4.6 ± 28.0
AS_L_	881.1 ± 101.7	57 ± 90.8	79.2 ± 5.3	4.2 ± 8.1	864.8 ± 34.5	27.3 ± 52.7
S_R_	117.5 ± 24.5	4.6 ± 14.4	34.4 ± 4.5	0.7 ± 5.6	120.4 ± 15.8	2.5 ± 19.6
D_R_	446.5 ± 34.5	13.9 ± 30.9	16.3 ± 2.8	0.2 ± 4.5	456.0 ± 18.2	1.3 ± 29.3
AS_R_	864.6 ± 106.4	38.7 ± 91.4	78.0 ± 7.1	1.4 ± 9.1	857.0 ± 46.2	9.1 ± 59.2
C_1_	42.3 ± 11.2	0 ± 9.8	12.7 ± 2.6	0.1 ± 3.5	44.5 ± 9.1	0.4 ± 12.3
C_2_	133.8 ± 15.3	−0.2 ± 14.8	40.2 ± 5.7	0.9 ± 5.4	140.7 ± 20.0	3.2 ± 18.9
C_3_	391.2 ± 48.9	7.2 ± 35.9	9.3 ± 2.5	0.6 ± 4.1	410.5 ± 16.3	3.9 ± 26.7
MID						
S_L_	55.6 ± 10.3	−1.1 ± 6.6	17.1 ± 1.8	−1.29 ± 2.19	59.9 ± 6.3	−4.5 ± 7.7
D_L_	461.2 ± 39.7	20.6 ± 30.4	20.5 ± 3.7	−0.54 ± 3.42	483.3 ± 24.1	−3.5 ± 22.2
AS_L_	858.5 ± 137.5	26.6 ± 103.9	79.9 ± 4.7	−0.17 ± 3.94	869.4 ± 30.6	−1.1 ± 25.6
S_R_	108.5 ± 18.0	−7.1 ± 24.1	35.3 ± 4.6	−3.93 ± 7.83	123.6 ± 16.1	−13.8 ± 27.4
D_R_	457.7 ± 68.5	26.3 ± 35.7	21.1 ± 3.1	0.20 ± 3.28	487.2 ± 20.2	1.3 ± 21.3
AS_R_	865.7 ± 129.3	25.4 ± 88.2	80.9 ± 4.6	−0.46 ± 1.67	875.9 ± 29.9	3.0 ± 10.9
C_1_	46.8 ± 12.6	2.4 ± 10.2	13.9 ± 2.8	0.26 ± 2.71	48.7 ± 9.8	0.9 ± 9.5
C_2_	135.0 ± 19.5	−2.8 ± 13.6	42.1 ± 6.1	−2.72 ± 2.17	147.4 ± 21.4	−9.5 ± 7.6
APEX						
S_L_	50.7 ± 9.73	−1.6 ± 11.5	14.7 ± 2.2	−1.0 ± 2.4	51.5 ± 7.7	−3.5 ± 8.4
D_L_	454.3 ± 28.6	14.6 ± 24.8	15.3 ± 6.3	0.3 ± 3.8	449.5 ± 41.0	2.0 ± 24.7
AS_L_	876.2 ± 105.2*	37.6 ± 83.7**	78.3 ± 4.7*	3.1 ± 5.1 **	859.0 ± 30.6	20.2 ± 33.2
S_R_	110.2 ± 20.6	−1.6 ± 19.8	32.8 ± 6.3	−2.2 ± 5.3	114.8 ± 22.1	−7.7 ± 18.6
D_R_	484.8 ± 43.6	8.0 ± 15.6	20.4 ± 4.7	−0.6 ± 4.4	482.6 ± 30.6	−3.9 ± 28.6
AS_R_	884.1 ± 106.1	14.4 ± 76.6	81.6 ± .4.4	0.8 ± 6.1	880.4 ± 28.6	5.2 ± 39.7
C_1_	49.4 ± 11.2	2.2 ± 10.8	13.8 ± 1.8	−0.1 ± 3.6	48.3 ± 6.3	−0.4 ± 12.6
C_2_	138.3 ± 21.0	−3.2 ± 12.6	40.5 ± 6.4	3.5 ± 6.3	141.8 ± 41.6	12.3 ± 22.1
C_3_	364.3 ± 38.0	10.7 ± 14.6	1.4 ± 3.3	−0.4 ± 5.6	359.1 ± 21.5	−2.6 ± 36.4

TTP values are shown as absolute times from the R wave (in ms) as well as percentages of systole or diastole as appropriate. For the latter, TTPs are also expressed in ms after normalising to a fixed systolic (350 ms) and diastolic length (650 ms). All values shown are mean ± standard deviation.

*1 excluded, **2 excluded since AS peak not clearly seen.

#### Regional variation

**Figure 4:**
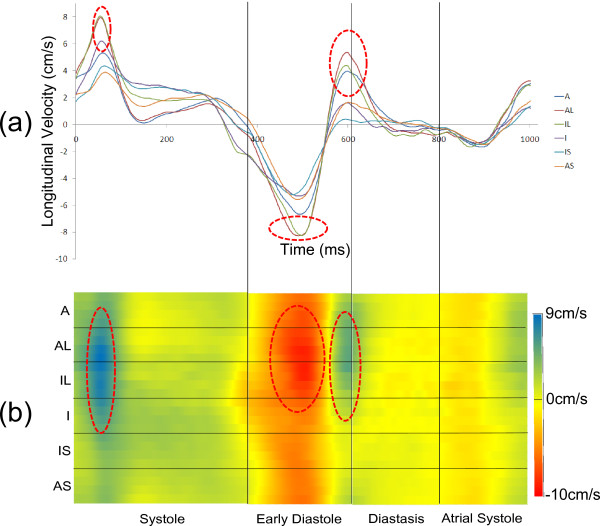
**Comparison of line plots and colour plots of the same longitudinal velocity data (a) Regional longitudinal mid-ventricular velocity-time curves averaged over all volunteers.** (**b**) The corresponding 2D colour plot showing regional variation. The positions of end systole, the beginning of diastasis and the beginning of atrial systole are marked by vertical lines. The horizontal lines split the colour plot into 6 regions to make comparison with the velocity-time curves easier. The red dotted circles highlight various regional features of motion and allow comparison of the velocity-time curves and 2D colour plot.

Average longitudinal regional velocity-time curves (over all 10 subjects) for the 6 AHA segments of the mid short-axis slice are shown in Figure 4(a). In order to correct for heart rate variations between subjects and to avoid the temporal smearing of peaks that this would cause – particularly in diastole - the data was normalised to a fixed RR interval prior to averaging. Rather than scaling the whole time-curve by a constant factor, it was separately normalised (using piecewise cubic interpolation) in four clearly defined sections: systole, early diastole, diastasis and atrial systole, as shown in Figure 4(a). Regional variations are apparent throughout the cardiac cycle, particularly at the times highlighted in the Figure by red dotted circles.

The same data can also be shown in a 2D colour plot (Figure 4(b)), allowing the visualisation of a finer segmentation (24 segments) of the myocardium. The vertical axis shows the segmental position around the entire circumference of the myocardium and the time evolution of its velocity is colour coded and plotted on the horizontal axis. The dotted circles in Figure 4(a) match those on the colour-plots to give reference points for interpretation. For example, the curves show that the highest peak diastolic velocities are in the lateral regions, but this is much more immediately obvious from the colour plots. The entire cardiac cycle is clearly displayed and regional differences in velocities and also TTP velocities are easily seen.

**Figure 5:**
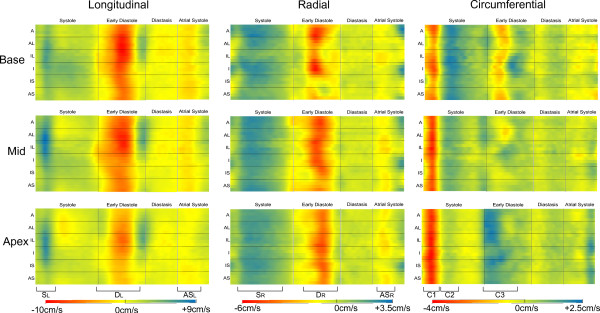
**2D colour plots averaged over all healthy volunteers for all three slices and directions.** In each case the positions of the various stages of motion are marked by vertical lines and the 6 AHA regions are marked by horizontal lines. The peaks referred to for global motion are indicated at the bottom of the figure. Each direction of motion has a separate scale as shown on the right hand side. Each direction of motion has a separate scale as shown beneath.

Figure 5 shows average normalised 2D colour plots for the basal, mid and apical short-axis slices in the longitudinal, radial and circumferential directions and enable rapid interpretation of a large amount of velocity data. Complicated patterns of motion can be seen in all slices and in all directions, as discussed below. By using the same colour scale for all three short-axis slices, the trend for increasing longitudinal velocities from apex to base, particularly for D_L_, can clearly be seen. Regional differences in peak and TTP values are obvious: for example the systolic peak S_L_ is strongest in the lateral regions in the basal and mid slices, whereas at apex it is strongest in the inferior segments, this change being indicated by a shift of the blue region vertically downwards on the apical colour plot when compared to the basal and mid plots. The atrial systolic peak is seen clearly at base and mid level and is less obvious at apical level.

Radial velocities show greater regional variation at base than at mid or apex. In particular the main diastolic peak D_R_ is strongest in the anterior and inferior regions at base, with the septum showing a biphasic pattern as previously described [16]. The atrial systolic peak AS_R_ is strongest in the inferolateral area at all levels.

Circumferential velocities all show a strong negative peak (C1) immediately after the R-wave. At base and mid levels this first peak is followed by a second peak in the opposite direction (C2) (which is strongest at base). C2 is also present at apical levels, although it can be either positive or negative, depending on the exact slice position. During early diastole the motion is highly dependent on slice position. At basal level, C3 is a sharp negative peak which shows some regional variation in timing, whereas at apex the peak is broader but positive, leading to the characteristic wringing motion of the heart. C3 is not present in the global velocity-time curves of the mid slice, however a small negative peak with highly regional timing can be seen on the colour plots in early diastole.

#### Transmural variation

**Figure 6:**
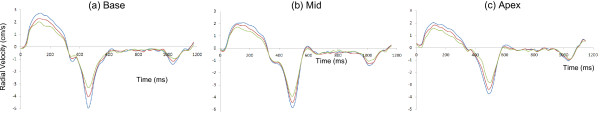
**Transmural radial velocities from the basal (a), mid (b) and apical (c) slice of a single healthy volunteer.** Transmural differences in radial velocity can clearly be seen for all peaks with epicardium higher than mid myocardium and mid myocardium higher than endocardium. The peaks are less obvious for longitudinal and circumferential velocities.

Table 3 shows global peak velocity values (mean ± SD averaged over all subjects) in the endocardium, mid-myocardium and epicardium in the mid short-axis slice. Highly significant differences in peak velocities are observed in the radial direction where peak systolic and diastolic endocardial velocities are significantly higher than peak mid-wall velocities (P < 0.001) which are in turn significantly higher than peak epicardial velocities (P < 0.001). Peak atrial systole velocities also show the same trend (P < 0.05). Example radial velocity-time curves demonstrating transmural velocity differences in all slices can be seen in Figure 6.

**Table 3:** Mean (± SD) peak endocardial, mid-myocardial and epicardial velocities for the mid short-axis slice of 10 healthy volunteers, together with the significance levels between them

**Peak velocity(cm/s)**	**Endocardium**	**Mid**	**Epicardium**	**P(Endo-Mid)**	**P(Mid-Epi)**
S_R_	2.75 ± 0.44	2.38 ± 0.41	2.09 ± 0.33	P < 0.001	P < 0.001
D_R_	−4.53 ± 0.74	−4.11 ± 0.61	−3.63 ± 0.51	P < 0.001	P < 0.001
AS_R_	−1.72 ± 0.43	−1.62 ± 0.45	−1.46 ± 0.41	P < 0.05	P < 0.01
S_L_	5.86 ± 1.94	5.92 ± 1.89	5.73 ± 1.89	NS	P < 0.05
D_L_	−6.67 ± 1.91	−6.72 ± 2.01	−6.60 ± 2.02	NS	P < 0.05
AS_L_	−1.81 ± 0.68	−1.87 ± 0.71	−1.99 ± 0.64	NS	NS
C_1_	−3.45 ± 1.48	−3.60 ± 1.52	−3.56 ± 1.57	P < 0.05	NS
C_2_	1.26 ± 0.76	1.35 ± 0.81	1.52 ± 0.85	P < 0.05	P < 0.001

Longitudinal peak velocities show no difference between endocardium and mid myocardium, however for both systolic and diastolic peaks there is a significant difference between mid-wall and epicardium (P = <0.05 for both). Circumferential velocities show significant differences in C1 between endocardium and mid (P < 0.05) and between all layers for C2 (P < 0.05 and P < 0.001 respectively).

### Reproducibility

The inter-study reproducibilities of retrospective spiral phase velocity mapping for measuring peak systolic and diastolic velocities are shown in Table 1. All global peak velocities have high reproducibility with the standard deviation of the signed differences ranging from 0.67 cm/s (apical S_L_) to 1.54 cm/s (mid D_L_) in the longitudinal direction, 0.27 cm/s (basal S_R_) to 0.96 cm/s (basal D_R_) in the radial direction and 0.38 cm/s (Apical C_2_) to 0.64 cm/s (basal C_3_) in the circumferential direction. Peak atrial systolic velocities also show small standard deviations of signed differences, particularly in the radial direction (0.41 cm/s (basal), 0.43 cm/s (mid) and 0.45 cm/s (apical)). E/A is more reproducible in the radial direction than in the longitudinal direction at all levels.

The inter-study reproducibilities of TTPs are shown in Table 2. The reproducibility of TTP (ms) of the systolic parameters (S_L_, S_R_, C1 and C2) is high at all slice levels (eg at mid level S_L_ = −1.1 ± 6.6 ms, S_R_ = −7.1 ± 24.1 ms, C_1_ = 2.4 ± 10.2 ms and C_2_ = −2.8 ± 13.6 ms), while that of later peaks (diastole and atrial systole) is reduced. When expressed in terms of percentages of systole or diastole as appropriate, the reproducibility is high for all TTP values. This improvement can best be seen by converting the percentages of systole or diastole into milliseconds based on the average length of systole or diastole: these values are shown in the last two columns of Table 2.

## Discussion

An efficient method for acquiring three directional velocities over the entire cardiac cycle using spiral k-space trajectories has been developed and is capable of acquiring high resolution images in a relatively short time. The implementation of retrospective cardiac gating has enabled analysis of myocardial mechanics through the entire cardiac cycle, including atrial systole which has not previously been seen with MR PVM techniques. We have presented global, regional and transmural data in the basal, mid and apical short-axis slices of 10 healthy volunteers together with a detailed analysis of the inter study reproducibility of systolic, early diastolic and late-diastolic peaks and their timings.

The use of spirals has allowed the acquisition of images with high spatial (1.4 mm × 1.4 mm) and temporal (21 ms) resolution in a short time (53 cardiac cycles) which compares favourably with previous Cartesian schemes (eg 2.6 × 1.3 mm, 13.8 ms (with view-sharing) in 128 cardiac cycles [16] or 2.6 × 1.4 mm, 26 ms in 180 cardiac cycles [19]). Spiral imaging can suffer from an increased level of artefact when compared with Cartesian imaging [20]. However, because of the short duration of spirals used in this study, and because of the careful attention to shimming, image quality has been very good, despite the high field strength (3 T). While off resonance artefact may sometimes be seen in regions of high susceptibility change, such artefacts are well localised and do not appear to interfere with the resulting velocity maps. An example of this type of artefact can be seen in the liver in Figure 2 close to the inferior regions of the LV.

A previous study found that simple accept/reject navigator algorithms using a single navigator per cardiac cycle are not sufficient to deal with respiratory motion for prospectively gated PVM of the myocardium [15]. Similarly preliminary data for this study found that these simple methods allow significant motion of the diaphragm to occur while data is acquired, thereby corrupting the velocities measured. By including the restriction that navigators both before and after the data is acquired had to be within the acceptance window, respiratory motion was better suppressed. Despite the necessity to have these strict navigator acceptance criteria the technique was efficient (average 57%) since the volunteers were able to guide their own breathing and most chose to complete the scan in a series of breath-holds rather than by regular slow breathing. However difficulties are expected when using this technique to image patients due to the level of compliance needed to use the respiratory trace to guide their own breathing. More sophisticated biofeedback mechanisms could be implemented in future patient studies [34].

During navigator output and feedback, no image data may be acquired. For prospective cardiac gating this means that no velocity information is available during this time, whereas for retrospective gating it results in decreased accuracy in the retrospective data interpolation close to the navigator. In this study, the navigator duration (9 ms) and feedback time (10 ms) were reduced as much as possible to minimise this problem. However, the retrospective cardiac gating allows us to calculate average velocities for the entire cardiac cycle, and, because they are close to zero (see Results), we can be confident that the accuracy of the technique overall is very good. An acceptance window of 5 mm has been used – as is conventional in high resolution coronary artery studies - which allows good suppression of respiratory artefact while ensuring that the scan efficiency is not too low.

Global longitudinal peak systolic and early diastolic velocities show the expected reductions from base to apex and this pattern is also seen in the atrial systolic peak velocities. The atrial systolic peak in longitudinal velocity measures the passive motion of the ventricle as the atria contract, pulling the base of the ventricle towards them. The peak velocity is therefore larger at base (−2.62 ± 0.99 cm/s) than at apex (−0.81 ± 0.29 cm/s) and was not seen in 3 (out of the 20) apical acquisitions, perhaps because atrial systole is not sufficiently strong in some patients to allow passive longitudinal motion to be detected at the apical level. Atrial systole also causes passive radial motion in the left ventricle at all levels, caused by blood being forced into the LV by the contraction of the atria: reduced radial peak velocity in atrial systole could therefore indicate reduced LV compliance. As expected, in the radial direction, there were no changes in the magnitude of any of the peak velocities from base to apex while in the circumferential direction, the characteristic wringing of the heart was clearly apparent through the opposite polarity of the C3 peaks at the basal and apical levels. The circumferential motion is most complicated and agrees well with previously published results [17].

In addition to the quantification of peak velocities, the retrospective cardiac gating has enabled us to determine the ratio of peak diastolic velocity and peak atrial systolic velocity (here called E/A) in healthy subjects. In echocardiography blood flow velocities at the mitral valve are measured at early diastolic filling (the E-wave) and during atrial systole (the A-wave). The blood velocity is well established to reflect the LA-LV pressure gradient and hence these measurements can give important information about preload, alterations in LV relaxation, LV compliance and LA contractile function: mitral annular velocities are also used to give similar information [32]. The ratio of E/A myocardial velocities could give similar information, helping our understanding of age-related changes of motion as well as pathological motion.

Time to peak (TTP) velocities have also been investigated and normal values are shown in Figure 3 and Table 2, both as measured from the R-wave and when normalised to a fixed systolic or diastolic length, as appropriate (expressed both in milliseconds and as a percentage). The latter method greatly reduces the variability of the TTP values between subjects, particularly in early and late diastole.

The high spatial resolution of our technique also allows detailed analysis of regional variations of myocardial motion. Regional myocardial velocity information has been displayed on colour plots. These are analogous to bulls eye plots which show all of the velocity information for the complete myocardium at a single time point in the cardiac cycle [35], but instead show the regional velocities for a given myocardial slice at all times in the cardiac cycle. This is an effective method of visualising regional variations in all velocity peaks and their timings. Average normal subject plots have been presented and could act as a reference for comparison with the diseased state. Prior to averaging, individual velocity-time curves were normalised to a fixed RR interval. While initial attempts at normalising to a fixed systolic and a fixed diastolic length reduced the blurring of peaks that would be apparent due to heart rate variations between subjects, peak preservation was further improved by normalising each time curve to fixed lengths of systole, early diastole, diastasis and atrial systole. The myocardium is typically 1 cm thick, so that there were approximately 7 pixels across the myocardial wall (14 reconstructed pixels). This allowed the analysis of transmural gradients, with significant differences between endocardial, mid myocardial and epicardial layers being detected.

**Figure 7:**
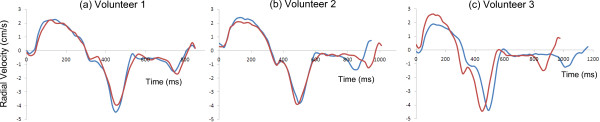
**Global radial velocities from the mid slices of three healthy volunteers on both occasions.** Volunteer 1 (**a**) showed similar heart rates on the two occasions and the two curves matchvery closely. However volunteers 2 (**b**) and 3 (**c**) showed different heart rates on the two occasions. TTP systole is not affected much by this change in either volunteer, however the TTP diastole is affected in Volunteer 3 but not in Volunteer 2. TTP atrial systole is affected in both volunteers. This inter-volunteer variation in the effects of different heart-rates makes the assessment of reproducibility more difficult.

**Figure 8:**
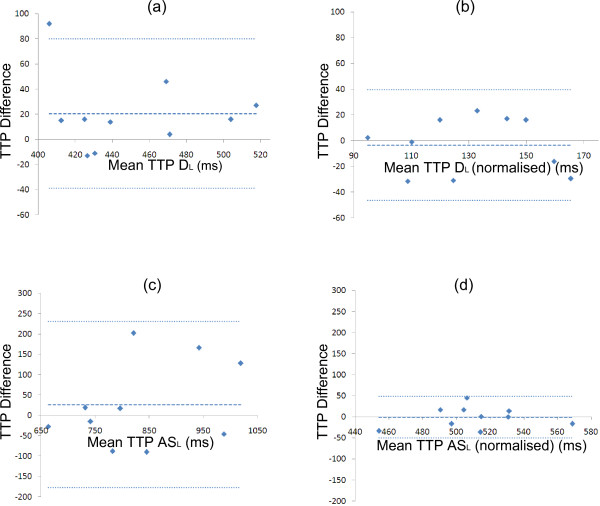
**Bland Altman plots showing the effect of taking the duration of diastole (normalising) into account on the reproducibility of TTP values.** (**a**) and (**b**) show the effect on TTP D_L_ whereas (**c**) and (**d**) show the effect on TTP AS_L_. In each case each blue diamond represents a single healthy volunteer with its position on the horizontal axis indicating the mean value of the two measurements of TTP and its position on the vertical axis indicating the difference between the two measurements. The blue dotted horizontal lines indicate the mean and ±2 standard deviations of the differences between the two measurements. For the diastolic and atrial systolic peaks the reproducibility is greatly improved by taking the length of diastole into account, showing that it is an effective way of compensating for healthy changes in heart rate between scans.

The inter-study reproducibilities of peak velocity data are high and are better than those obtained with lower temporal resolution sequences, both using breath-holding techniques (eg S_R_ in the mid slice was −0.01 ± 0.36 cm/s compared with −0.12 ± 0.84 cm/s with 37–87 ms temporal resolution [14]) and navigator techniques (eg −0.56 ± 0.87 cm/s for mid D_L_ compared with −0.51 ± 2.1 cm/s in a navigator study with 35 ms resolution [36]). The inter-study reproducibility of TTP (as measured from the R wave) is strongly dependent on heart rate, particularly for the diastolic peaks. This is clear from the example radial velocity plots in the mid slice of three volunteers from acquisitions performed in two separate days which are shown in Figure 7. In volunteer 1, the heart rate was the same on both occasions and the velocity-time curves agree well. In volunteer 2, the RR interval changed by approximately 50 ms between the two acquisitions and, while the systolic and early diastolic portions of the curves agree well, the length of diastasis is different and the atrial peaks therefore do not coincide. In the third volunteer, the RR intervals changed by approximately 200 ms affecting the timing of all major peaks. By normalising to the length of systole or diastole [14], the reproducibility of TTP measurements is greatly improved. This is demonstrated by the Bland Altman plots in Figure 8 where the interstudy reproducibility (SD of the mean differences between acquisition repeats) is reduced from 30 ms to 22 ms for the early diastolic peak and from 104 ms to 26 ms for the atrial systolic peak. Expressing TTP values relative to systolic or diastolic length is therefore clearly preferable to absolute measurements from the R wave.

A Cartesian trajectory PVM technique has been used previously to assess healthy motion in subjects and in several patients [18,35], however long scan times and incomplete measurement of the cardiac cycle, as well as restricted information on the reproducibility of the measurements, have so far limited the application of this technique. We have presented a more efficient method of collecting this data and have comprehensively assessed its reproducibility. A full analysis of the impact of using spiral trajectories rather than Cartesian, and of using retrospective instead of prospective cardiac gating will be carried out as future work. Images acquired at 3 T have better SNR than they would at lower field strengths and so there is the potential to further speed up the acquisition [37] by using non-Cartesian parallel imaging such as SENSE [38]. This would also allow the development of a breath-hold version of the sequence with better temporal and spatial resolution than has previously been possible. Currently the imaged slices are fixed in space. Slice following could be introduced to improve the technique. The time consuming manual delineation of the myocardium is also a limitation, but automatic or semi-automatic segmentation algorithms could improve this in the future.

## Conclusions

By using spiral k-space trajectories, high temporal and spatial resolution PVM images can be acquired in a much reduced time when compared with conventional imaging techniques. Fine regional and temporal variation in velocities can be seen, and retrospective cardiac gating enables the assessment of atrial systole. The 2D colour plots clearly and effectively depict regional velocities allowing easy assessment of myocardial motion over the entire cardiac cycle. The reproducibility of the technique has been comprehensively assessed. Spiral PVM has the potential to be a useful and reproducible clinical tool for assessing regional myocardial motion.

## Abbreviations

CMR: Cardiovascular magnetic resonance; DENSE: Displacement encoding with stimulated echoes; LV: Left ventricle; PVM: Phase velocity mapping; SD: Standard deviation; SENC: Strain encoded imaging; SNR: Signal to noise; TTP: Time to peak.

## Competing interests

The authors declare that they have no competing interests.

## Authors’ contributions

RS was involved in developing the sequence, collecting and analysing the data and drafting the manuscript. JK was involved in designing the study, developing the sequence and revising the manuscript. DF was involved in designing the study and revising the manuscript. All authors have read and approved the final manuscript.

## Authors’ information

Jennifer Keegan and David Firmin are joint senior authors.
